# Advisory Committee on Immunization Practices Recommended Immunization Schedule for Children and Adolescents Aged 18 Years or Younger — United States, 2020

**DOI:** 10.15585/mmwr.mm6905a3

**Published:** 2020-02-07

**Authors:** Candice L. Robinson, Henry Bernstein, Katherine Poehling, José R. Romero, Peter Szilagyi

**Affiliations:** ^1^Immunization Services Division, National Center for Immunization and Respiratory Diseases, CDC; ^2^Zucker School of Medicine at Hofstra/Northwell and Cohen Children’s Medical Center, New Hyde Park, New York; ^3^Wake Forest School of Medicine/Wake Forest Baptist Health and Brenner Children’s Hospital, Winston-Salem, North Carolina; ^4^University of Arkansas for Medical Sciences and Arkansas Children's Hospital, Little Rock, Arkansas; ^5^Department of Pediatrics, University of California Los Angeles, Los Angeles, California.

At its October 2019 meeting, the Advisory Committee on Immunization Practices (ACIP)* approved the 2020 Recommended Child and Adolescent Immunization Schedule for Ages 18 Years or Younger. The 2020 child and adolescent immunization schedule summarizes ACIP recommendations, including several changes from the 2019 immunization schedule^†^ on the cover page, three tables, and notes found on the CDC immunization schedule website (https://www.cdc.gov/vaccines/schedules/index.html). Health care providers are advised to use the tables and the notes together. This immunization schedule is recommended by ACIP (https://www.cdc.gov/vaccines/acip/index.html) and approved by the CDC Director, the American Academy of Pediatrics, the American Academy of Family Physicians, the American College of Obstetricians and Gynecologists, and, for the first time, the American College of Nurse-Midwives.

ACIP’s recommendations on use of each vaccine are developed after in-depth reviews of vaccine-related data, including the epidemiology and burden of the vaccine-preventable disease, vaccine efficacy and effectiveness, vaccine safety, quality of evidence, feasibility of program implementation, and economic analyses of immunization policy (*1*). The child and adolescent immunization schedule is published annually to consolidate and summarize updates to ACIP recommendations on vaccination of children and adolescents and to assist health care providers in implementing current ACIP recommendations. The use of vaccine trade names in this report and in the child and adolescent immunization schedule is for identification purposes only and does not imply endorsement by ACIP or CDC.

For further guidance on the use of each vaccine, including contraindications and precautions, health care providers are referred to the respective ACIP vaccine recommendations at https://www.cdc.gov/vaccines/hcp/acip-recs/index.html. Providers should be aware that changes in recommendations for specific vaccines can occur between annual updates to the child and adolescent immunization schedule. If errors or omissions are discovered within the child and adolescent schedule, CDC posts revised versions on the CDC immunization schedule website.^§^ Printable versions of the 2020 child and adolescent immunization schedule and ordering instructions are available on the immunization schedule website at https://www.cdc.gov/vaccines/schedules/hcp/imz/child-adolescent.html.

## Changes in the 2020 Child and Adolescent Immunization Schedule

Changes in the 2020 child and adolescent immunization schedule for persons aged 18 years or younger include new or updated ACIP recommendations for hepatitis A vaccine (HepA) (*2*); influenza vaccine (*3*); meningococcal B vaccine (MenB) (*2*); and tetanus toxoid, reduced diphtheria toxoid, and acellular pertussis vaccine (Tdap) (*4*). Changes also include clarification of the recommendations for diphtheria and tetanus toxoids and acellular pertussis vaccine (DTaP), *Haemophilus influenzae* type b vaccine (Hib), hepatitis B vaccine (HepB), meningococcal ACWY vaccine (MenACWY), and poliovirus vaccine. Following are the changes to the cover page, Tables 1–3, and the Vaccine Notes.

**Cover page**

- The American College of Nurse-Midwives has been added to the list of organizations that approve the child and adolescent immunization schedule.
- Trademark symbols (®) were added to all vaccine trade names.

**Table 1**

- **HepA row:** The bar for persons aged 2–18 years has been changed to solid green to denote the recommendation for routine catch-up immunization for all persons in this age group.
- **HPV row:** An asterisk has been added to the blue bar that appears for children aged 9–10 years to indicate that for this group, the HPV vaccine series can be started at the clinician’s discretion. The text that defines the blue box in the table’s legend has been edited and now reads “Recommended based on shared clinical decision-making or *can be used in this age group.”
- **Legend:** The text that defines the gray box has been edited and now reads “No recommendation/not applicable.”

**Table 2**

- **Meningococcal rows:** The letters “ACWY” were added to clarify that these catch-up intervals apply only to MenACWY and not to MenB.

**Table 3**

- **Hep A row:** All boxes now appear yellow to denote the recommendation for routine vaccination for all persons aged 18 years or younger, including those with the medical indications outlined in the table.
- **MenACWY row:** The pregnancy box is now yellow, because the meningococcal vaccine may be administered to pregnant women, if indicated.
- **Legend:** The text that defines the red box has been edited and now reads “Not recommended/contraindicated—vaccine should not be administered.” The text that defines the gray box has been edited and now reads “No recommendation/not applicable.”

**Vaccine Notes**

- **DTaP:** To clarify the recommendations for catch-up vaccination, the note has been updated to indicate that dose 5 is not necessary if dose 4 was administered at age 4 years or older AND at least 6 months after dose 3.
- **Hib:** A bullet has been added to note that catch-up vaccination is not recommended for previously unvaccinated children aged 5 years (60 months) or older who are not at high risk.
- **HepA:** The note was revised to include the recommendation that all children and adolescents aged 2 through 18 years who have not previously received Hep A should receive catch-up vaccination and complete a 2-dose series.
- **HepB:** A “Special situations” section has been added which contains information regarding populations for whom revaccination might be recommended. The ACIP HepB recommendations are referenced for detailed revaccination recommendations.
- **Influenza vaccine:** The note has been updated to reflect the recommendations for the 2019–20 influenza season. The “Routine vaccination” section was reformatted to more clearly outline circumstances under which 1 or 2 doses of influenza vaccine are recommended. In addition, the bullet that outlines circumstances under which live attenuated influenza vaccine (LAIV) should not be used was reformatted into a bulleted list.
- **MenACWY:** Guidance regarding adolescent vaccination for children who received MenACWY before age 10 years has been added to the note.
- **MenB:** Booster doses are now recommended for persons aged ≥10 years with complement deficiency, those who use complement inhibitors, persons with asplenia, persons who are microbiologists, and persons determined by public health officials to be at increased risk during an outbreak. The MenB note has been updated to include a link to the detailed recommendations.
- **Poliovirus vaccination**: Detailed information has been added regarding which oral poliovirus vaccine (OPV) doses may be counted toward the U.S. vaccination requirements.
- **Tdap:** The note has been updated to allow either Td or Tdap, as an option for decennial tetanus booster doses and catch-up series doses in persons who have previously received Tdap. In addition, the note has been edited to reflect recent updates to the clinical guidance for children aged 7 through 18 years who received doses of Tdap or DTaP at age 7 through 10 years. A dose of Tdap or DTaP administered at age 10 years may now be counted as the adolescent Tdap booster. A dose of Tdap or DTaP administered at age 7 through 9 years should not be counted as the adolescent dose, and Tdap should be administered at age 11–12 years.

## Additional Information

The Recommended Child and Adolescent Immunization Schedule, United States, 2020 is available at https://www.cdc.gov/vaccines/schedules/hcp/imz/child-adolescent.html. The full ACIP recommendations for each vaccine are also available at https://www.cdc.gov/vaccines/hcp/acip-recs/index.html. All vaccines identified in Tables 1, 2, and 3 (except DTaP, rotavirus, and poliovirus vaccines) also appear in the Recommended Adult Immunization Schedule for Ages 19 Years or Older, United States, 2020.^¶^ The notes for vaccines that appear in both the adult immunization schedule and the child and adolescent immunization schedule have been harmonized to the greatest extent possible.

## Footnotes

* Recommendations for routine use of vaccines in children and adolescents are developed by the Advisory Committee on Immunization Practices (ACIP), a federal advisory committee chartered to provide expert external advice and guidance to the CDC Director on use of vaccines and related agents for the control of vaccine-preventable diseases in the civilian population of the United States. Recommendations for routine use of vaccines in children and adolescents are harmonized to the greatest extent possible with recommendations made by the American Academy of Pediatrics (AAP), the American Academy of Family Physicians (AAFP), the American College of Obstetricians and Gynecologists (ACOG), and the American College of Nurse-Midwives (ACNM). ACIP recommendations approved by the CDC Director become agency guidelines on the date published in the *Morbidity and Mortality Weekly Report* (*MMWR*). Additional information about ACIP is available at https://www.cdc.gov/vaccines/acip.

† Past immunization schedules are available at https://www.cdc.gov/vaccines/schedules/past.html.

§ CDC encourages organizations to use syndication as a more reliable method for displaying the most current and accurate immunization schedules on an organization's website rather than copying these schedules to their websites. Use of content syndication requires a one-time step that ensures an organization’s website displays current schedules as soon as they are published or revised; instructions for the syndication code are available on CDC’s website (https://www.cdc.gov/vaccines/schedules/syndicate.html). CDC also offers technical assistance for implementing this form of content syndication (e-mail request to ncirdwebteam@cdc.gov). Information on changes in ACIP recommendations in the child and adolescent immunization schedule before the next scheduled annual update, if any, is available at https://www.cdc.gov/vaccines/schedules/hcp/schedule-changes.html#child.

¶ https://www.cdc.gov/vaccines/schedules/hcp/imz/adult.html.
